# Yeast mitochondrial HMG proteins: DNA-binding properties of the most evolutionarily divergent component of mitochondrial nucleoids

**DOI:** 10.1042/BSR20150275

**Published:** 2016-01-22

**Authors:** Jana Bakkaiova, Victoria Marini, Smaranda Willcox, Jozef Nosek, Jack D. Griffith, Lumir Krejci, Lubomir Tomaska

**Affiliations:** * Departments of Genetics and Biochemistry, Comenius University in Bratislava, Faculty of Natural Sciences, Mlynska dolina, Ilkovicova 6, 842 15 Bratislava, Slovak Republic; † Department of Biology, Masaryk University, Kamenice 5/A7, Brno 625 00, Czech Republic; ‡ International Clinical Research Center, St. Anne's University Hospital in Brno, Brno 60200, Czech Republic; § Lineberger Comprehensive Cancer Center, University of North Carolina at Chapel Hill, NC 27599, U.S.A.; ║ National Center for Biomolecular Research, Masaryk University, Kamenice 5/A7, Brno 625 00, Czech Republic

**Keywords:** DNA-binding protein, DNA compaction, HMG-box containing protein, Holliday junction, mitochondrial DNA (mtDNA), mitochondrial nucleoid

## Abstract

Yeast mtDNA is compacted into nucleoprotein structures called mitochondrial nucleoids (mt-nucleoids). The principal mediators of nucleoid formation are mitochondrial high-mobility group (HMG)-box containing (mtHMG) proteins. Although these proteins are some of the fastest evolving components of mt-nucleoids, it is not known whether the divergence of mtHMG proteins on the level of their amino acid sequences is accompanied by diversification of their biochemical properties. In the present study we performed a comparative biochemical analysis of yeast mtHMG proteins from *Saccharomyces cerevisiae* (*Sc*Abf2p), *Yarrowia lipolytica* (*Yl*Mhb1p) and *Candida parapsilosis* (*Cp*Gcf1p). We found that all three proteins exhibit relatively weak binding to intact dsDNA. In fact, *Sc*Abf2p and *Yl*Mhb1p bind quantitatively to this substrate only at very high protein to DNA ratios and *Cp*Gcf1p shows only negligible binding to dsDNA. In contrast, the proteins exhibit much higher preference for recombination intermediates such as Holliday junctions (HJ) and replication forks (RF). Therefore, we hypothesize that the roles of the yeast mtHMG proteins in maintenance and compaction of mtDNA *in vivo* are in large part mediated by their binding to recombination/replication intermediates. We also speculate that the distinct biochemical properties of *Cp*Gcf1p may represent one of the prerequisites for frequent evolutionary tinkering with the form of the mitochondrial genome in the CTG-clade of hemiascomycetous yeast species.

## Abstract (precis)

Comparative biochemical analysis of mtHMG proteins from distantly related yeast species revealed that they exhibit a preference for recombination/replication intermediates. We discuss how these biochemical characteristics relate to the role of mtHMG proteins in mtDNA compaction and evolution.

## INTRODUCTION

The compaction of DNA into chromosomes enables not only its accommodation into the confines of the cell, but also provides a means for spatial regulation of gene expression and protection from DNA damage. Given its importance, it is not surprising that the compaction of DNA into nucleosomes is mediated by a complex of highly conserved proteins called histones [1]. Their extremely high level of conservation is exemplified by the fact that only eight of 102 amino acids differ between the H4 histones of such evolutionary distant species as humans and *Saccharomyces cerevisiae* [2]. A deficiency in a histone-encoding gene, or even an imbalance in their expression is often fatal, or can accelerate aging [3,4], further underlining the importance of these proteins in mediating principal functions in DNA maintenance and gene expression [5].

The necessity for DNA compaction is not limited to the eukaryotic nucleus, as it also extends to bacterial cells as well as DNA-containing organelles, namely chloroplasts and mitochondria. In the latter, the DNA is compacted into nucleoprotein structures called mitochondrial nucleoids (mt-nucleoids) [6–11]. This compaction is in part mediated by proteins containing two DNA-binding domains known as an high-mobility group (HMG)-box [12]. The best-characterized members of this group of proteins (mitochondrial HMG-box containing proteins; mtHMG proteins) are *Sc*Abf2p [13–15] and mammalian mitochondrial transcription factor A (TFAM) [16–20]. Apparently, during the evolution of eukaryotic cells, the host genome-encoded mtHMG proteins replaced the polypeptides that served as the major nucleoid components in the original α-proteobacterial endosymbiont [21]. Yet, although it seems that the presence of an HMG-box is a universal feature of mitochondrial compaction proteins in all eukaryotes, their overall amino acid sequences exhibit very low similarity. In fact, mtHMG proteins seem to represent one of the most divergent groups of mitochondrial proteins [22]. Thus, in contrast with histones, it is very difficult to identify them by simple bioinformatic tools and most of the mtHMG proteins were identified using proteomic analyses of purified mt-nucleoids [22–25].

The dissimilarities between mtHMG proteins can be explained either by the fast evolutionary divergence of the common ancestor or by acquisition of new features. Perhaps the heterogeneity at the level of amino acid sequences in the mtHMG proteins corresponds to different biochemical roles in mtDNA maintenance and segregation, or possibly differences in mtDNA base composition and topology. To explore this question we have performed a comparative biochemical analysis of mtHMG proteins from distantly related species. For this purpose, yeast species from the subphylum Saccharomycotina represent an ideal group of organisms. They exhibit a high degree of biodiversity [26], their mtDNAs differ in size, base composition and topology [27], and mtHMG proteins were identified in a number of species separated by hundreds of millions of years of evolution [15,22–24,28–30]. However, the only biochemically characterized yeast mtHMG protein is Abf2 of *S. cerevisiae* [13–15,31–35]. It was shown that *Sc*Abf2p prefers negatively supercoiled DNA over circular or linear DNA and that, in cooperation with a DNA topoisomerase, it introduces negative supercoils into a topologically relaxed, covalently closed circular dsDNA molecule [15,32]. Its binding to mtDNA is nonrandom, which may be accomplished by the phased distribution of short stretches of poly(dA) indicating its role in genome organization and site-specific regulation of transcription or DNA replication [32]. Optical trapping of single DNA molecules extended by flow and visualized by fluorescence microscopy allowed determination of the binding constant of Abf2 (*K*_b_=2.57±0.74×10^7^ M^−1^) [31] (but see also [35]), and relative small forces (<0.6 pN) stabilizing the condensed DNA–protein interactions [31]. AFM revealed that at high concentrations of Abf2, the DNA is compacted into relatively loosely packaged 190 nm structures [31,33] indicating that Abf2 is indeed the bona fide mtDNA-packaging protein. This conclusion was also reached based on the results of an *in organello* ChIP-on-chip assay demonstrating that *Sc*Abf2p binds to most of the mitochondrial genome with a preference for GC-rich gene sequences [36].

Although these studies provided important information about the DNA-binding properties of *Sc*Abf2, they also left several important questions unanswered. First, the *in vitro* studies were mostly performed on intact dsDNA substrates, whereas yeast mitochondria contain topologically different forms of DNA generated as a result of various types of transactions including replication, recombination and repair [37–40]. It is known that HMG-box containing proteins recognize some of these structures with high affinity [12]. However, information about the binding of yeast mtHMG proteins to DNA substrates such as Holliday junctions (HJ) or replication forks (RF) is lacking. Second, the compaction of DNA is induced at relatively high *Sc*Abf2p to DNA-binding site ratios (20 to 1) [31,33], whereas in the organelle there is one molecule of *Sc*Abf2p per 27 bp of DNA (size of the binding site) [15] (see also below). And third, basically all biochemical data on mtHMG proteins are derived from the studies of *Sc*Abf2p. As indicated above, the mtHMG proteins represent the fastest evolving component of mt-nucleoids [22] and it is currently unknown if this divergence in amino acid sequence translates into differences in biochemical properties.

**Figure 1:**
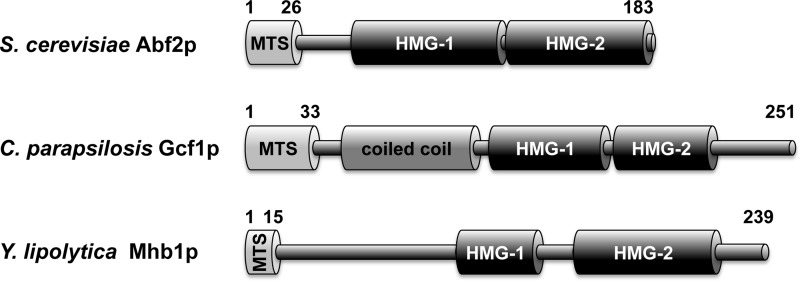
Yeast mtHMG proteins Domain prediction in Abf2, Gcf1 and Mhb1 proteins is based on previous reports [15,22,23,30]. MTS–mitochondrial targeting sequence (not present in the mature protein). Note that although SMART (http://smart.embl-heidelberg.de) and InterProScan (http://www.ebi.ac.uk/interpro/) searches did not identify HMG-1 box in *Cp*Gcf1p, the corresponding region appears to be weakly conserved with HMG-1 box detected in its orthologues from *Candida lusitaniae*, *Candida subhashii*, *Debaryomyces hansenii* and *Meyerozyma guilliermondii*.

To address these questions we have performed a comparative analysis of mtHMG proteins from three distinct yeast species (Figure 1). Namely, we selected *Sc*Abf2p from *S. cerevisiae* as the best-characterized protein thus allowing comparison of our results with those published by other authors. *Yl*Mhb1p from *Yarrowia lipolytica* [23] was chosen because this species belongs to basal lineages of Saccharomycotina and phylogenetically is very distant to *S. cerevisiae*. Therefore, we could compare biochemical properties of two proteins with very divergent amino acid sequences, whose only common feature is the presence of two HMG-boxes. Finally, *Cp*Gcf1p is the mtHMG protein from *Candida parapsilosis*, the yeast species with a linear mitochondrial genome [22,41]. A detailed characterization of this protein enabled us to address the question of how biochemical properties of mtHMG proteins may be associated with the evolutionary emergence of the linear mitochondrial genome forms, frequently occurring in species from the CTG-clade of Saccharomycotina encompassing *C. parapsilosis*.

## MATERIALS AND METHODS

### Microbial strains and growth conditions

*Escherichia coli* DH5α (F^−^, *φ80dlacZΔM15, Δ(lacZYA-argF) U169, deoR, recA1, endA1, hsdR17 (rk^−^, mk^+^), λ, thi-1, gyrA96, relA1, glnV44, nupG*) (Life Technologies) was used for the amplification of plasmid constructs. *E. coli* BL21 Star™(DE3) (*F^−^, ompt, hsdSB, rB^−^mB^−^, gal, dcm, rne131*) (Life Technologies) was used for production of recombinant proteins (*Sc*Abf2noMP, *Yl*Mhb1noMP, *Cp*Gcf1noMP). Bacterial cultures were grown in LB medium (1% (w/v) bacto peptone (Difco), 0.5% (w/v) yeast extract (Difco), 1% (w/v) NaCl, pH 7.5) containing 100 μg/ml ampicillin.

### DNA manipulations

Enzymatic manipulations with DNA, cloning procedures and DNA labelling were performed according to the instructions provided by the vendors. The oligonucleotides (Table 1) were synthesized by Microsynth. The PCRs were performed in 10–50 μl volumes using Dream*Taq* DNA polymerase (Life Technologies) or Phusion Hot Start II High fidelity DNA polymerase (Life Technologies) and contained all four dNTPs (final concentration 200 μM each), the corresponding primers (final concentra-tion 1 μM), and either 100 ng of genomic DNA or mtDNA or 10 ng of plasmid DNA. The PCR fragments were purified from agarose gels using a QIAquick Gel Extraction kit (Qiagen) or Zymoclean Gel DNA recovery kit (Zymo Research).

**Table 1:** List of oligonucleotides *1*, preparation of pGEX-6P-2 derived vectors. *2*, preparation of DNA substrates used in EMSA experiments.

Name	sequence 5'→3'	Application
ScABF2noMP_F	AAGGCTTCCAAGAGAACGCAGC	*1*
YlMHB1noMP_F	AAGGAGGCTGCCACTAAGACC	*1*
pGEX6P2noMP_R	GGGCCCCTGGAACAGAACTT	*1*
CpGCF1noMP_F	TCAACCGCCAAAACCACTC	*1*
CpGCF1noMP_R	TTAGATTGTGAATTTGTACTCTTGT	*1*
ScATP9_15_D	GGAGCAGGTATTGGT	*2*
ScATP9_15_C	ACCAATACCTGCTCC	*2*
ScATP9_25_D	GGAGCAGGTATTGGTATTGCTATCG	*2*
ScATP9_25_C	CGATAGCAATACCAATACCTGCTCC	*2*
ScATP9_50_R	ACACCATTAATTAAAGCTGC	*2*

### Construction of plasmid vectors

For the expression of recombinant versions of mtHMG proteins lacking the cleavable mitochondrial import sequence (noMP) in fusion with GST a series of pGEX-6P-2 (GE Healthcare) derived plasmids was constructed as follows. Plasmids pGEX-6P-2-*ScABF2*noMP and pGEX-6P-2-*YlMHB1*noMP were prepared by inverse PCR to eliminate the first 26 amino acids for *Sc*Abf2p and 14 amino acids for *Yl*Mhb1p, corresponding to cleavable mitochondrial import sequence. In the reaction, primers pGEX6P2noMP_R and ScABF2noMP_F or YlMHB1noMP_F (Table 1) were used and previously prepared plasmids pGEX-6P-2-*ScABF2* or pGEX-6P-2-*YlMHB1* [23] were used as templates. For the construction of the plasmid pGEX-6P-2-*CpGCF1*noMP the *CpGCF1* ORF lacking the first 33 amino acids, which represent cleavable mitochondrial import sequence, was amplified by PCR from the genomic DNA of *C. parapsilosis* strain CBS604 using primers CpGCF1noMP_F and CpGCF1noMP_R (Table 1). The PCR product was inserted into the vector pGEX-6P-2 (GE Healthcare) linearized with SmaI. All plasmid constructs were verified by restriction enzyme mapping and DNA sequencing (Microsynth) of the inserted fragments.

### Expression and purification of recombinant mtHMGp from *E. coli*

Recombinant mtHMG proteins without the mitochondrial import presequence were purified from bacterial cells as described previously for full length *Yl*Mhb1 protein [23]. The presence and purity of proteins were verified by 12% SDS-PAGE stained with Coomassie Brilliant Blue R-250.

### DNA substrates and electrophoretic-mobility shift assay (EMSA)

**Figure 2:**
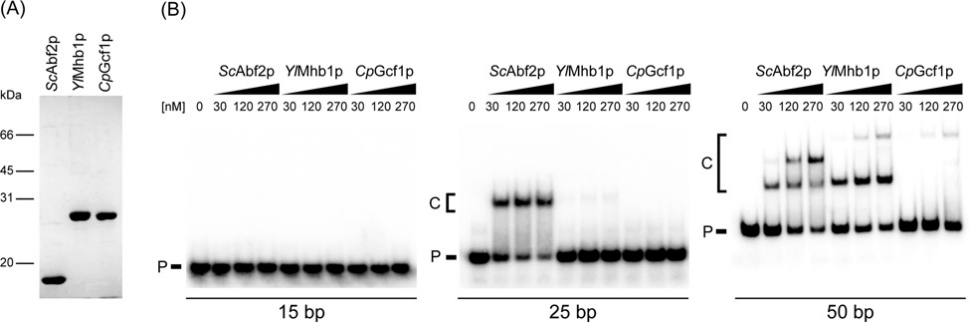
Purified yeast mtHMG proteins differ in their ability to bind dsDNA of various lengths (**A**) Purified mtHMG proteins were visualized by Coomassie staining of gels after their separation by 12% SDS-PAGE. (**B**) The proteins (at indicated concentrations) were incubated with 3 nM of 15 bp, 25 bp or 50 bp radioactively labelled dsDNA substrates and the samples were separated in polyacrylamide gels as described in Materials and methods. P, free DNA probe; C, DNA–protein complexes.

For electrophoretic-mobility shift assay (EMSA) experiments aimed at characterizing the length of the binding site for mtHMG proteins (Figure 2) oligonucleotides ScATP9_15_D and ScATP9_25_D (Table 1), derived from the *S. cerevisiae* mitochondrial gene *atp9*, were radioactively labelled using T4 polynucleotide kinase (Life Technologies) and [γ^32^P]ATP. The labelled oligonucleotide was then mixed with non-labelled complementary oligonucleotide (ScATP9_15_C or ScATP9_25_C, Table 1) in a molar ratio of 1:3. The mixtures were incubated at 95°C for 5 min and cooled slowly to room temperature to allow DNA annealing. The unincorporated [γ^32^P]ATP was removed from the DNA by gel filtration using Probe Quant G-50 MicroColumns (GE Healthcare). The 50 bp long DNA substrate derived from the *atp9* gene was amplified by PCR from mtDNA of *S. cerevisiae* strain W303-1A using primers ScATP9_15_D and ScATP9_50_R (Table 1) and terminally end-labelled using T4 polynucleotide kinase. Fluorescently labelled DNA substrates for EMSA were prepared as described previously [42,43]. The structures of the DNA probes are schematically depicted in the corresponding figures. The GC content of the probes ranged between 40% (50 bp probe) and 53% (15 bp probe), which is higher than the GC content in *atp9* coding sequence (33%).

Indicated amounts of purified recombinant proteins *Sc*Abf2noMP, *Yl*Mhb1noMP or *Cp*Gcf1noMP were mixed with the individual radioactively or fluorescently labelled DNA substrate (3 nM) and incubated for 10 min at 30°C in 10 μl of a buffer containing 20 mM Tris/HCl pH 7.5, 1 mM EDTA/NaOH pH 8.0, 50 mM NaCl, 100 μg/ml BSA. Samples were electrophoretically separated in 5 or 8% (w/v) polyacrylamide gels in 0.5× TBE buffer (45 mM Tris/borate, 1 mM EDTA/NaOH pH 8.0) at 4°C. Note that the loading buffer contained only a final concentration of 5% (v/v) glycerol, as we observed that the presence of bromophenol blue and xylene cyanol blue almost completely abolished the binding of mtHMG proteins to DNA. Radioactively labelled DNA substrates were visualized after exposing the gels to storage phosphor screens (Kodak) for 24–72 h using a Personal Molecular Imager FX (BioRad). Fluorescent DNA substrates were visualized directly using imager reader FLA-9000 Starion (Fuji) and quantified using MultiGauge V3.2 software (Fuji).

### Electron microscopy

The DNA-binding reactions for EM were performed in 50 μl of HEN buffer (20 mM HEPES/NaOH pH 7.5, 1 mM EDTA/NaOH pH 8.0, 50 mM NaCl) containing 2 ng/μl of the substrate DNA and 15 ng/μl of purified *Sc*Abf2p. Plasmid pGLGAP and preparation of the RF and HJ substrates were described elsewhere [44,45]. The reactions were carried out at room temperature for 15 min, followed by addition of 10 μl of 1.2% (v/v) glutaraldehyde and incubation at room temperature for additional 6 min. To remove the unbound proteins and fixative, the samples were diluted to 50 μl in HEN buffer and passed over 2 ml columns of 6% agarose beads (ABT Inc.) equilibrated with TE buffer (10 mM Tris/HCl, pH 7.4, 0.1 mM EDTA/NaOH). Aliquots of the fractions containing the complexes were mixed with a buffer containing spermidine and adsorbed on to copper grids coated with a thin carbon film glow-charged shortly before sample application. Following adsorption of the samples for 3 min, the grids were dehydrated through a graded ethanol series and rotary shadowcast with tungsten at 10^−7^ torr [46]. Samples were examined in an FEI T12 TEM equipped with a Gatan 2k × 2k SC200 CCD camera.

## RESULTS

### Yeast mtHMG proteins differ in their affinity to dsDNA

To initiate the biochemical characterization of mtHMG proteins, we expressed recombinant genes encoding the corresponding protein without the N-terminal mitochondrial targeting sequence in fusion with GST using the pGEX-6P-2 vector. To obtain native versions of the proteins, the fusion proteins purified from *E. coli* were treated with *PreScission* protease to remove the GST affinity tag (Figure 2A). The concentrations of the proteins were then adjusted to the same value and their affinity to dsDNA assessed by EMSA.

The proteins were first tested for their ability to bind dsDNA of various lengths (15–50 bp) derived from the *S. cerevisiae atp9* gene (Figure 2B). Although none of the proteins was able to bind the shortest (15 bp) DNA fragment, the 25 bp DNA was almost quantitatively shifted by *Sc*Abf2p. *Yl*Mhb1p exhibited very weak binding and *Cp*Gcf1p did not bind this probe at all. The 50 bp DNA fragment was bound by all three mtHMG proteins. Both *Sc*Abf2p and *Yl*Mhb1p formed two DNA–protein complexes, possibly corresponding to one and two protein molecules per molecule of DNA, respectively. *Cp*Gcf1p formed only a single complex with DNA, although its mobility corresponded to the slower migrating form of DNA bound by *Yl*Mhb1p. Although the nature of this complex is unclear (see Discussion), it is evident that the affinities of the mtHMG proteins to dsDNA as well as the lengths of their corresponding DNA-binding sites differ, and that the *Cp*Gcf1p exhibits the lowest affinity towards intact dsDNA.

### ScAbf2p exhibits a high preference for replication/recombination intermediates

The results presented in Figure 2B indicate that to obtain quantitative binding of mtHMG proteins to dsDNA, even in the case of *Sc*Abf2p (which exhibits the strongest binding), a relatively high protein to DNA-binding site ratio (50–100 to 1) is required. A relatively high ratio of *Sc*Abf2p to binding sites was also required for a complete compaction of DNA by *Sc*Abf2p as visualized by AFM [31,33]. Namely, to completely compact a linear DNA (pBR322) containing 175 binding sites, almost 4000 molecules of *Sc*Abf2p were needed (a ratio of 22 *Sc*Abf2p to 1 DNA-binding site) [31], and similar results were obtained by another group [33]. At such high ratios of protein to DNA, contamination of the native protein with denatured molecules or truncated species, either of which may induce a general aggregation or collapse of the DNA, becomes a significant concern.

**Figure 3:**
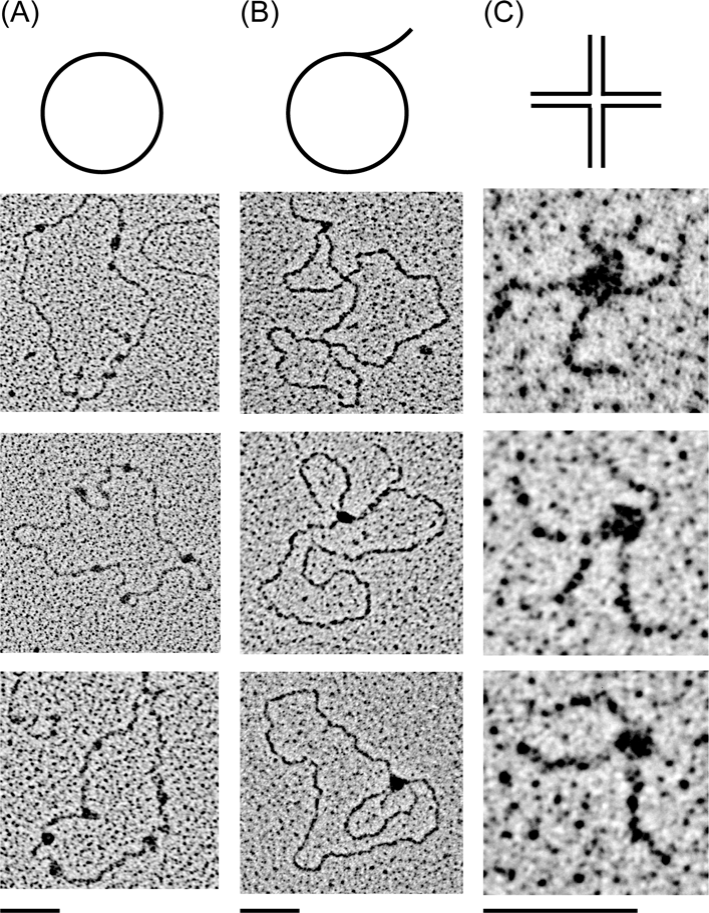
*Sc*Abf2 protein exhibits a binding preference for RF and HJ 15 ng/μl of purified *Sc*Abf2p were incubated with 2 ng/μl of pGLGAP (**A**) RF (**B**) or HJ (**C**) substrates for 15 min at room temperature. The samples for EM were prepared as described in Materials and methods. The bars represent 50 nm.

*In vivo*, the ratio of *Sc*Abf2p to DNA is much lower than what has been employed in the previous biochemical studies. Diffley and Stillman [15] originally estimated the amount of *Sc*Abf2p to be 250000 molecules per cell, but more recent assessments of the cellular amount of Abf2p resulted in two orders of magnitude lower numbers, namely 3810 [47] and 860 [48] molecules of Abf2p per cell, respectively. If there are 50–100 molecules of 85 kbp mtDNA per cell and the length of the binding site is 27 bp (Figure 2B; [15]), there would be 160000–320000 binding sites per cell and thus even when the highest estimate of Abf2p molecules is taken into account, the stoichiometric ratio of *Sc*Abf2p to DNA substrate *in vivo* is 1 to 1 (when considering the lower estimates, the ratio would drop dramatically). Under these conditions, the binding of *Sc*Abf2p to dsDNA is 3–5-fold lower (Figure 2B) and the level of compaction is almost negligible [31,33]. Indeed, when we visualized the binding of *Sc*Abf2p to plasmid DNA by EM at a protein to DNA-binding site ratio of 1 to 1 we observed only 5–10 protein particles per DNA molecule (Figure 3A). Therefore, we reasoned that intact dsDNA may not be the best substrate for this protein. Rather, *Sc*Abf2 may prefer DNA forms resulting from various types of DNA transactions such as replication and recombination. This hypothesis was supported by the fact that many nuclear mtHMG proteins exhibit a preference for recombination intermediates [12] and yeast mitochondria possess a DNA recombination system involved in both DNA replication and repair [49]. We therefore tested the ability of *Sc*Abf2p to bind RF or HJ by EM. Indeed, we found that the binding of protein to DNA occurred almost exclusively at the RF (Figure 3B) or at the junction (Figure 3C). These results prompted us to compare the binding of all three mtHMG proteins to various DNA substrates by EMSA.

### Comparative analysis of yeast mtHMG proteins reveals differences in their binding preferences to various DNA substrates

**Figure 4:**
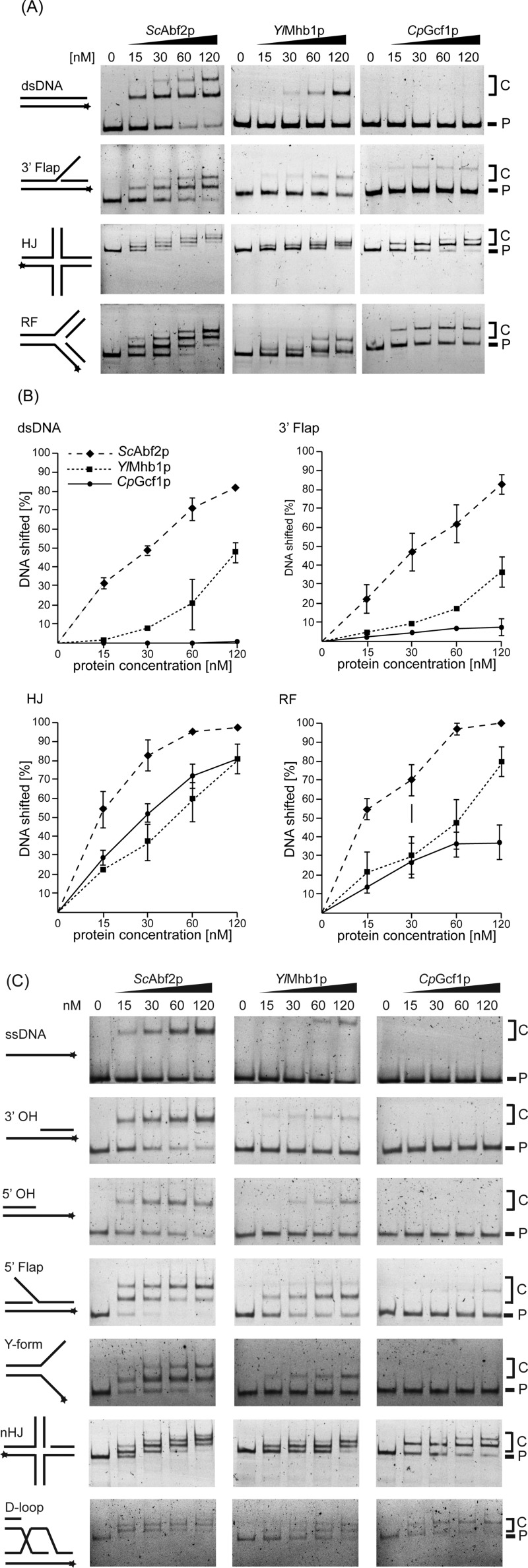
EMSA of yeast mtHMG proteins with various DNA substrates reveals both similarities and differences in their DNA-binding properties (**A**) The proteins (at indicated concentrations) were incubated with 3 nM of the fluorescently labelled DNA substrates (dsDNA, 3′ Flap, HJ and RF DNA), whose predicted structures are indicated on the left side of each panel. Stars indicate the position of the fluorescent dye. P, free probe; C, DNA–protein complexes. (**B**) The percentage of the shifted DNA fragments was quantified using Multi Gauge V3.2 software (Fuji). The results represent an average of at least three independent experiments. (**C**) Analysis of the binding of yeast mtHMG proteins to ssDNA, 3′ OH, 5′ OH, 5′ Flap, Y-form, nHJ and D-loop DNA was performed as in Figure 4A. P, free probe; C, DNA–protein complexes.

To compare the DNA-binding properties of the mtHMG proteins we tested 10 different DNA substrates, namely intact dsDNA (see also Figure 2B), HJ, RF, 3′ Flap, 5′ Flap, single-strand (ss), 3′ and 5′ overhang (OH), Y-form, nicked and intact HJ (nHJ a HJ) and displacement loop (D-loop) DNAs (Figure 4). The similarities and differences between the proteins are best illustrated by the first four substrates (Figures 4A and 4B). *Sc*Abf2p exhibited the strongest binding to each of these structures with intact dsDNA and the 3′ Flap substrates being the least preferred. The difference in binding between the three proteins was most evident on intact dsDNA. To shift 50% of the probe 30 nM of *Sc*Abf2p was sufficient, whereas 120 nM of *Yl*Mhb1p was needed, and *Cp*Gcf1p did not shift 50% of dsDNA even at the highest tested concentration (Figure 4B). On the other hand, RF containing DNA, and especially HJ DNAs were bound very efficiently by all three mtHMG proteins even at lower protein concentrations (Figures 4A and 4B). It is of note that *Cp*Gcf1p, which bound dsDNA and 3′ Flap substrates very poorly, was almost as efficient in binding to the HJ DNA as *Sc*Abf2p (Figure 4B). This demonstrates that the low affinity of *Cp*Gcf1p to dsDNA and 3′ Flap DNA is not a result of the protein being nonfunctional, but rather reflects its intrinsic biochemical preference for certain types of DNA substrates. The binding of the proteins to other DNA substrates, including ssDNA, 5′ Flap, Y-form, 3′ OH and 5′ OH substrates was similar as to dsDNA, whereas the nHJ and D-loop DNAs were bound as efficiently as the HJ DNA (Figure 4C).

**Figure 5:**
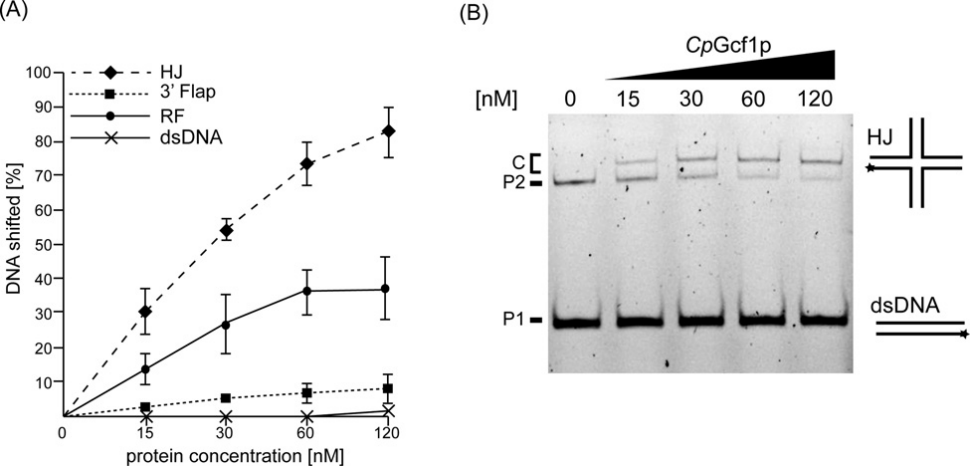
*Cp*Gcf1p exhibits the most dramatic difference in binding to dsDNA and 3′ Flap compared with HJ and RF substrates (**A**) Quantification of the binding of *Cp*Gcf1p to four different substrates using data from Figure 4. (**B**) *Cp*Gcf1p was incubated in the reaction mixture containing both dsDNA and HJ DNA and the samples were separated by electrophoresis in polyacrylamide gel as described in Materials and methods. P1, free dsDNA probe; P2, free HJ probe; C, DNA–protein complexes.

Intriguingly, *Cp*Gcf1p exhibits the largest difference in its ability to bind recombination intermediates (HJ, nHJ, D-loop DNAs) compared with dsDNA and RF DNA. At the protein concentration of 120 nM, *Cp*Gcf1p exhibits an 8-fold higher affinity towards HJ compared with 3′ Flap DNA (Figure 5A). Therefore, we have performed competition experiments, where the protein is incubated simultaneously with both dsDNA and HJ DNA. Whereas the former is basically unrecognized, there is a relatively robust binding of the protein to the HJ substrate (Figure 5B).

## DISCUSSION

Our results point out several important properties of yeast mtHMG proteins. First, intact dsDNA is a relatively poor substrate. *Sc*Abf2p exhibited the highest apparent affinity for dsDNA; however, to achieve a quantitative binding, high protein to DNA ratios were needed (Figure 2B). Whereas *Yl*Mhb1p exhibited similar binding to the 50 bp long dsDNA as *Sc*Abf2p, it bound very weakly to 25 bp dsDNA. This might be caused by a longer binding site of *Yl*Mhb1p compared with *Sc*Abf2p. The binding of *Cp*Gcf1p to dsDNA was very weak for all substrates even at the highest protein to DNA ratios (Figures 2B and 5). In case of the 50 bp probe the shift by the *Cp*Gcf1p corresponds to the supershift observed by *Yl*Mhb1p and *Sc*Abf2p suggesting that protein dimers are bound to the probe. The presence of a coiled-coil domain in *Cp*Gcf1p might be responsible for the formation of oligomeric complexes, but this possibility needs to be tested experimentally. The role of dimerization in DNA binding and compaction was studied in detail in case of the mammalian mtHMG protein (TFAM) and is still a matter of debate. Two crystal structures of TFAM bound to the heavy strand promoter or to a nonspecific mtDNA sequence showed evidence of TFAM dimerization [50]. On the other hand, a more recent study demonstrated that dimerization is not required for TFAM-induced compaction of mtDNA [51] underlining the importance of further investigation of interactions of mtHMG proteins with DNA.

Even though *Sc*Abf2p might bind to some sites on mtDNA with higher preference, it was found to bind to most of the genome *in vivo* [36]. Taking into account the ratio of *Sc*Abf2p to DNA *in vivo* (at most one molecule of the protein per one 27 bp binding site, but possibly much lower (see above)) our results indicate that the role of yeast mtHMG proteins in the compaction of mtDNA might not be as straightforward as suggested by previous studies. This conclusion is supported by the fact that cells lacking *Sc*Abf2p [9], *Yl*Mhb1p [23] or the Gcf1p orthologue of *Candida albicans* (*Ca*Gcf1p) [29], exhibit morphologically different, yet still functional mt-nucleoids. This can be explained by the existence of distinct mtDNA-compaction factors acting in parallel with the mtHMG proteins. Some of the candidates (such as Aco1p, Ilv5p) were identified in *S. cerevisiae* [7,52,53]; however, the means by which these proteins mediate compaction of mtDNA are far from understood.

The second general important property of the yeast mtHMG proteins we observed is their preference for DNA structures generated during replication, recombination and/or repair (Figures 3 and 4). This biochemical property is common for proteins of the HMG-1/HMG-2 subfamily that were shown to bind distorted structures such as HJ, cis-platinum adducts or base bulges [54]. This is also true for mammalian mtHMG protein TFAM that was demonstrated to exhibit 10-fold higher affinity for HJ [55] as well as RNA four-way junctions [56] compared with the linear dsDNA. Although it was shown that an R-loop formed in the human mitochondrial replication origin contains a Holliday-like structure [57], the level of DNA recombination in mammalian mitochondria is (at best) relatively low [58] and thus the physiological role of high-affinity binding of TFAM to HJ structures is not clear. A recent elegant study of Kukat et al. [51] revealed that TFAM-mediated nucleoid formation *in vitro* is a multistep process initiated by TFAM aggregation and cross-strand binding. It is possible that yeast mtHMG proteins also employ similar mechanisms of mtDNA compaction. However, in contrast with their mammalian counterparts, yeast mitochondria exhibit a high level of recombination DNA intermediates [37–40]. In fact, recombination seems to be a principal mechanism of yeast mtDNA replication [39]. The high incidence of recombination intermediates in yeast mitochondria combined with our results demonstrating a preference of yeast mtDNA proteins for such DNA structures indicate that these structures may represent their main substrates *in vivo*. Indeed, the *abf2*Δ mutants of *S. cerevisiae* [59,60] and knockdown *gcf1*^−^ strains of *C. albicans* [29] exhibit a decrease in the level of mtDNA recombination intermediates. Conversely, overexpression of the *ABF2* gene in *S. cerevisiae* results in an increased level of recombination intermediates and destabilization of mtDNA [59]. In addition, both *abf2*Δ and *mhb1*Δ mutants of *S. cerevisiae* and *Y. lipolytica*, respectively, are more prone to mutations [23,61]. This may be the result of both deprotection of mtDNA and/or a decreased capacity of recombination-dependent DNA repair. Although all these results indicate that binding of mtHMG proteins to various DNA structures is important for mtDNA transactions in yeasts, the molecular details of their participation in these processes are still largely unknown.

The observation of Kucej et al. [36] that *in vivo* Abf2p binds to most of the mitochondrial genome with a preference for GC-rich gene sequences can also be interpreted in light of our results. First, the ChIP-on-chip assay was performed on a population of cells and could not address the distribution of *Sc*Abf2p at the level of single mtDNA molecules. Second, GC-clusters are hot-spots of mtDNA recombination in *S. cerevisiae* mitochondria [62], so it is possible that the preferential binding to these regions was caused by recombination undergoing at these sites.

Taken together, it is likely that the compaction of mtDNA by yeast mtHMG proteins is achieved via a combination of cross-strand binding of intact DNA (as demonstrated for TFAM; [51]) and binding to DNA recombination/replication intermediates that are abundant in yeast mitochondria. It was shown that the nuclear protein *Sc*Ixr1, a paralogue of *Sc*Abf2p, binds and bends intrastrand DNA cross-links induced by platinum [63] and thus participates in the repair of these DNA lesions. When HJ or RF DNAs were used as substrates for mtHMG proteins, we observed the formation of several distinct species of DNA–protein complexes, some of which may contain oligomeric forms of the protein and/or compacted DNA (Figure 4). It is possible that these DNA structures serve as signals for binding of mtHMG proteins followed by their oligomerization accompanied by compaction of DNA and/or recruitment of other components of mt-nucleoids. Considering the high amount of recombination intermediates in yeast mitochondria, it would be interesting to investigate how they, in concert with mtHMG proteins, participate in the formation of mt-nucleoids and how the mode of binding of mtHMG proteins to various substrates is affected by interactions with other nucleoid-associated proteins, as well as their posttranslational modifications such as phosphorylation and proteolytic cleavage [64–66].

One of the main motivations for the present study was to address the question of whether the high degree of amino acid divergence among the yeast mtHMG proteins corresponds to differences in their biochemical properties. On one hand, the proteins seem to be similar in their ability to complement (although to a different extent) the *abf2Δ* mutation in *S. cerevisiae* [22,23]. Also, they all exhibit a preference for recombinational intermediates compared with intact dsDNA. On the other hand, their relative affinities for various substrates differ, especially when comparing *Sc*Abf2p and *Yl*Mhb1p with *Cp*Gcf1p. In contrast to its counterparts, *Cp*Gcf1p hardly binds dsDNA, whereas its binding to RF and especially HJ DNA is comparable to the other two mtHMG proteins (Figures 4A and 4B). Of note is the inability of *Cp*Gcf1p to bind to 5′ OH containing DNA under the conditions tested (Figure 4C) as this structure is present at the ends of linear mtDNA of *C. parapsilosis* [41] and therefore is relatively frequent *in vivo*. Apparently, the ss/double-stranded (ds) junction does not seem to be a preferred site for *Cp*Gcf1p. Moreover, the 5′ OH terminus of mtDNA of *C. parapsilosis* is covered by the mitochondrial telomere-binding protein (mtTBP) [67–69] and it is also engaged in the formation of telomeric loops (t-loops) [70], and thus the junction would probably not be accessible for binding *in vivo*.

Although, based on its biochemical properties, *Cp*Gcf1p does not seem to be present at the terminal regions of the linear mtDNA of *C. parapsilosis in vivo*, the dramatic differences between its ability to bind recombination intermediates (HJ, nHJ, D-loop DNA) compared with dsDNA and RF DNA (Figure 5A) indicate that it is an important player employed by *C. parapsilosis* mitochondria to maintain mtDNA in general and mitochondrial telomeres in particular. When analysed by 2D agarose electrophoresis, preparations of mtDNA of *C. parapsilosis* contain a large variety of recombination intermediates indicating that recombination plays an important part in mtDNA replication [40]. Moreover, maintenance of mitochondrial telomeres composed of tandemly repeated sequences is mediated by telomeric circles (t-circles), whose formation is dependent on the recombination machinery [71–73]. Their replication via a rolling-circle mechanism generates an array of telomeric sequences that can reintegrate back into the main mtDNA molecules via homologous recombination [71,73]. Moreover, we have shown previously that mtDNA of *C. parapsilosis* forms t-loops where 5′ OH invades into the ds region of the telomere [70]. All these features highlight a crucial role of *Cp*Gcf1p in DNA recombination and thus in the maintenance of *C. parapsilosis* mtDNA as well as its telomeres.

**Figure 6:**
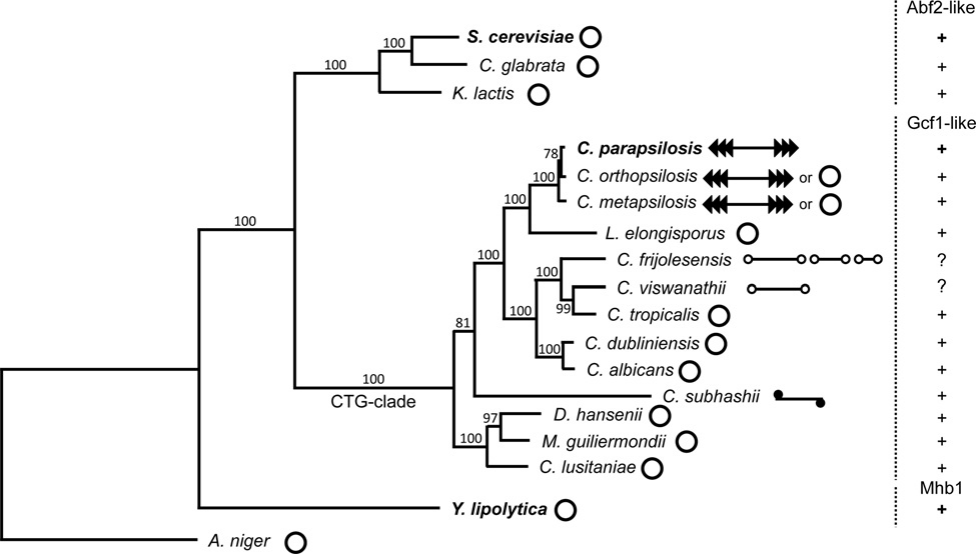
Phylogenetic tree illustrating the distribution of various types of mtHMG proteins and variability of the form of mitochondrial genomes in selected yeast species The phylogeny was calculated from concatenated multiple sequence alignments of mtDNA-encoded proteins (i.e. Atp6-8-9-Cob-Cox1-2-3-Nad1-2-3-4-4L-5-6-Rps3) by the maximum likelihood algorithm and LG (Le-Gascuel) amino acids substitution model implemented in the PhyML program [80]. Bootstrap values (out of 100 replicates) are shown above the corresponding branches. *Aspergillus niger* from the subphylum Pezizomycotina was used as an outgroup. Mitochondrial genome forms were classified as described previously [76] and are illustrated by pictograms (open circles–circular; lines with open circles at the ends–linear with terminal hairpins (i.e. type 1 linear and multipartite type 1 linear); lines with series of arrowheads–linear with array of tandem repeats (i.e. type 2 linear); line with closed circle at the ends–linear with a protein covalently bound to 5′ termini (i.e. type 3 linear). The species whose mtHMG proteins were investigated in the present study are shown in bold.

Finally, it is of note that *Sc*Abf2p and *Yl*Mhb1p are biochemically more similar to each other than to *Cp*Gcf1p, although *S. cerevisiae* and *Y. lipolytica* are phylogenetically more distant than either species is to *C. parapsilosis* (Figure 6). It is possible that this observation can be explained by differences in the evolutionary dynamics of mitochondrial genome architecture in the corresponding phylogenetic branches. Although *S. cerevisiae* and *Y. lipolytica* are separated by about 500 million years [26], they belong to groups of species mostly possessing a circular-mapping mitochondrial genome, whose mode of maintenance is likely quite similar. This would explain the similarity in biochemical properties of *Sc*Abf2p and *Yl*Mhb1p. On the other hand, *C. parapsilosis* is a member of a CTG-clade of the subphylum Saccharomycotina exhibiting a wide repertoire of forms of mitochondrial genomes ranging from circular-mapping to linear molecules with defined telomeres such as tandem repeats, hairpins, or covalently attached proteins [27,74–76]. The conversion between various forms seems to be quite frequent, often occurring in strains of the same species [77,78]. It is possible that distinct biochemical features of the Gcf1 protein described in the present study represent one of the prerequisites allowing such frequent evolutionary tinkering [79].

## Abbreviations

*Cp*: *Candida parapsilosis*
D-loop: displacement loop
ds: double-stranded
EMSA: electrophoretic-mobility shift assay
HJ: Holliday junctions
HMG: high-mobility group
mt: mitochondrial
nHJ: nicked Holliday junction
RF: replication forks
*Sc*: *Saccharomyces cerevisiae*
ss: single-stranded
TFAM: transcription factor A, mitochondrial
t-loop: telomeric loop
*Yl*: *Yarrowia lipolytica*
